# Supplementary material for: Deep reefs of the Great Barrier Reef offer limited thermal refuge during mass coral bleaching
Source: Nat Commun. 2018 Sep 4;9:3447. doi: 10.1038/s41467-018-05741-0 (PMC6123414; doi:10.1038/s41467-018-05741-0)
Supplement: Supplementary file 1 — Supplementary Information [file 41467_2018_5741_MOESM1_ESM.pdf]

Supplementary Information

Deep reefs of the Great Barrier Reef offer limited thermal refuge during mass coral bleaching

by Frade et al.

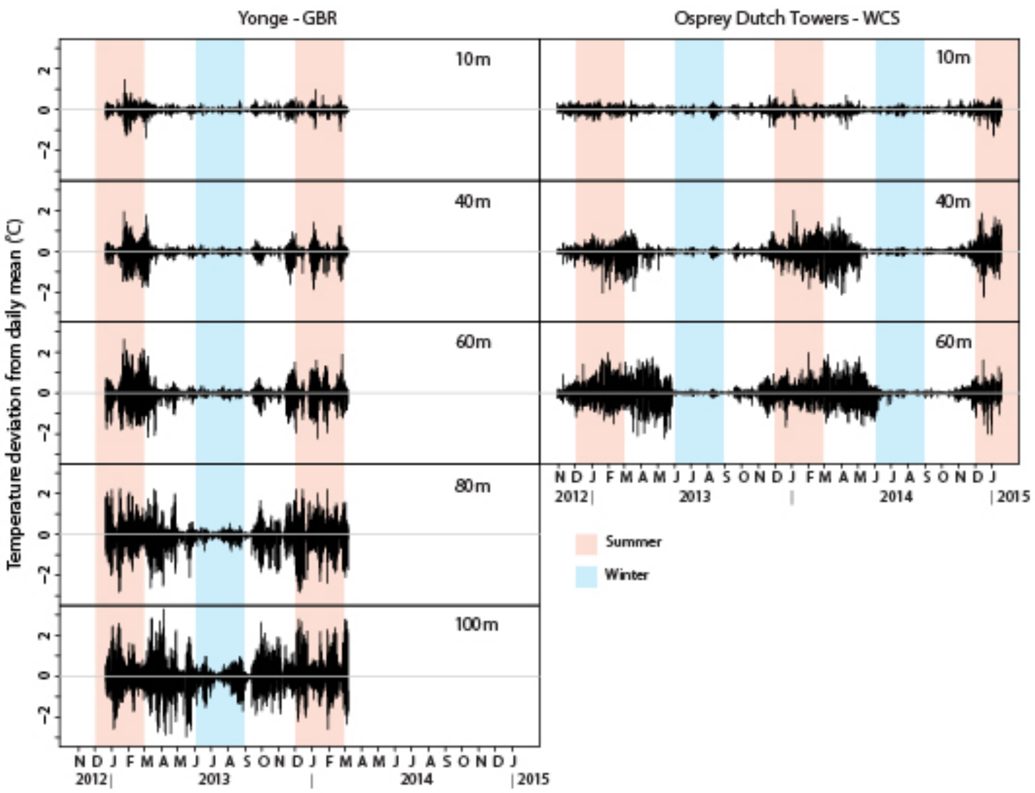

Supplementary Figure 1. Deviation from daily mean temperatures at 10-100 m depth at Yonge Reef on the Great Barrier Reef (GBR) and Osprey Reef in the Western Coral Sea (WCS). Panels, from top to bottom, refer to temperature deviation at 10, 40, 60, 80 and 100 m depth. Note that for Osprey data is unavailable for 80 and 100 m depth. Cold-water influxes at both the GBR and WCS sites result in average absolute deviations from the mean daily temperature ranging 0.1-0.2 °C at the shallows and upper mesophotic depths (10 and 40 m depth), and 0.2-0.5 °C at lower mesophotic depths (60-100 m depth), during the warm period of October 2013 – February 2014. Despite this similar range, weekly-monthly temperature oscillations at mesophotic depths appear more irregular at the GBR than the WCS site, likely the result of intermittent seasonal upwelling as well as the combined contribution of oceanic and tidal flow of the GBR lagoon water[1,2]. Due to these cold-water incursions from the deep at both GBR and WCS, the amount of time for which the temperature deviated more than 1 °C from the daily mean during the

summer months increased drastically from <1% of the time at 10 m and <2% at 40 m, up to 5-17% of the time at 100 m depth.

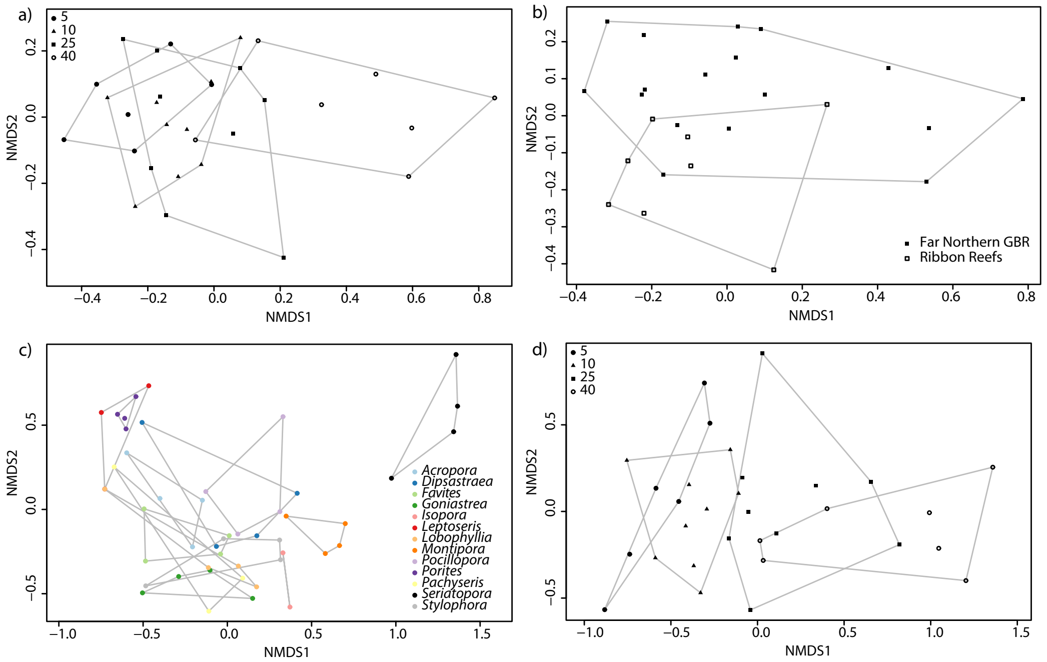

Supplementary Figure 2. Non-Metric Multidimensional Scaling (NMDS) ordination based on quantitative Bray-Curtis dissimilarities. NMDS panels depict **a)** the effect of depth, and **b)** the effect of region (Far Northern GBR versus Ribbon reefs) on the abundance distribution of the distinct bleaching categories (healthy, minor bleaching, severe bleaching and recently dead) for all coral taxa combined; **c)** the effect of taxonomic affiliation on the abundance distribution of the distinct bleaching categories for all locations combined; and **d)** the effect of depth on coral community structure (with genus-resolution) for all locations combined.

Community-wide effect of depth on bleaching distribution (panel a) was confirmed statistically by constrained ordination (CCA, $F_{(29,1)}$=16.293, p<0.01) and by multivariate analyses based on Bray-Curtis similarities (ANOSIM statistic R=0.238, p<0.001; and PERMANOVA pseudo $F_{(29,1)}$=18.549, p<0.001; results validated by the homogeneity of multivariate dispersions). Most of the dissimilarity between the deep reef (40 m) and the three shallow depths (5, 10 and 25 m) was caused by a statistically significant overrepresentation of healthy colonies at 40 m and of severely bleached colonies at 5-

25 m at the time of the survey, as well as an overrepresentation of minor bleaching at 5 m (SIMPER analysis).

Although the bleaching was widespread across the studied GBR locations[3], there was a tendency in the data to over-represent severely bleached colonies in the Ribbon region when compared to the far northern sites in the present study. This is demonstrated by a statistical effect of region (panel b: Ribbons vs Far Northern sites) on bleaching distribution (PERMANOVA pseudo $F_{(29,1)}$=3.7673, p<0.05, and CCA, $F_{(29,1)}$=4.9557, p<0.01). This geographical effect was not significant when analyzing only 10–40 m depth communities, showing that it is the 5 m community that decisively shapes this trend. This result supports previously published data on the geographical response of shallow reefs (as quantified by aerial surveys) to bleaching[3]. Unlike region, individual site/location had no significant effect on the distribution of the distinct bleaching categories.

Our statistical analyses (all locations combined) clearly show that bleaching response is a function of taxon affiliation (panel c; CCA, $F_{(35,12)}$=12.866, p<0.01; ANOSIM statistic R=0.5193, p<0.001; and PERMANOVA pseudo $F_{(35,12)}$=8.5891, p<0.001). These dissimilarities in bleaching distribution among coral taxa at the time of the survey are mostly driven (according to SIMPER) by an overrepresentation of healthy colonies in *Porites* and *Leptoseris* (concomitant with a lower than average abundance of severely bleached colonies), an overrepresentation of severely bleached colonies in *Montipora* and *Isopora*, and finally by a higher than average abundance of recently dead colonies in *Seriatopora* (concurrent with low abundance of slightly bleached colonies in this genus). For this dataset with all locations combined, depth is also a significant driver of the bleaching response, but as expected the depth effect is not homogeneous across species (interaction species : depth, results not shown).

Despite the demonstrated effect of depth on the incidence of bleaching at the community level, coral community composition was also driven by depth (panel d; CCA, $F_{(29,1)}$=9.9863, p<0.01; ANOSIM statistic R=0.3212, p<0.001; and PERMANOVA pseudo $F_{(29,1)}$=13.78, p<0.001), and, to a minor extent, by location

(results not shown). This depth divergence in community structure (panel d) was mostly driven by a higher abundance of *Favites* and *Isopora* at 5 m than on the deeper reef (10-40 m), as well as by a higher abundance of *Pachyseris*, *Seriatopora* and *Leptoseris* at 40 m than on the shallow reef (5-25 m). Because different coral taxa have different bleaching susceptibilities (see panel c) due to physiological properties inherent to the coral animal itself or to its symbiotic communities[4,5], this means that at least part of the relief in bleaching incidence offered by the deep reef may actually be explained by differences in community composition (see Supplementary Figure 3).

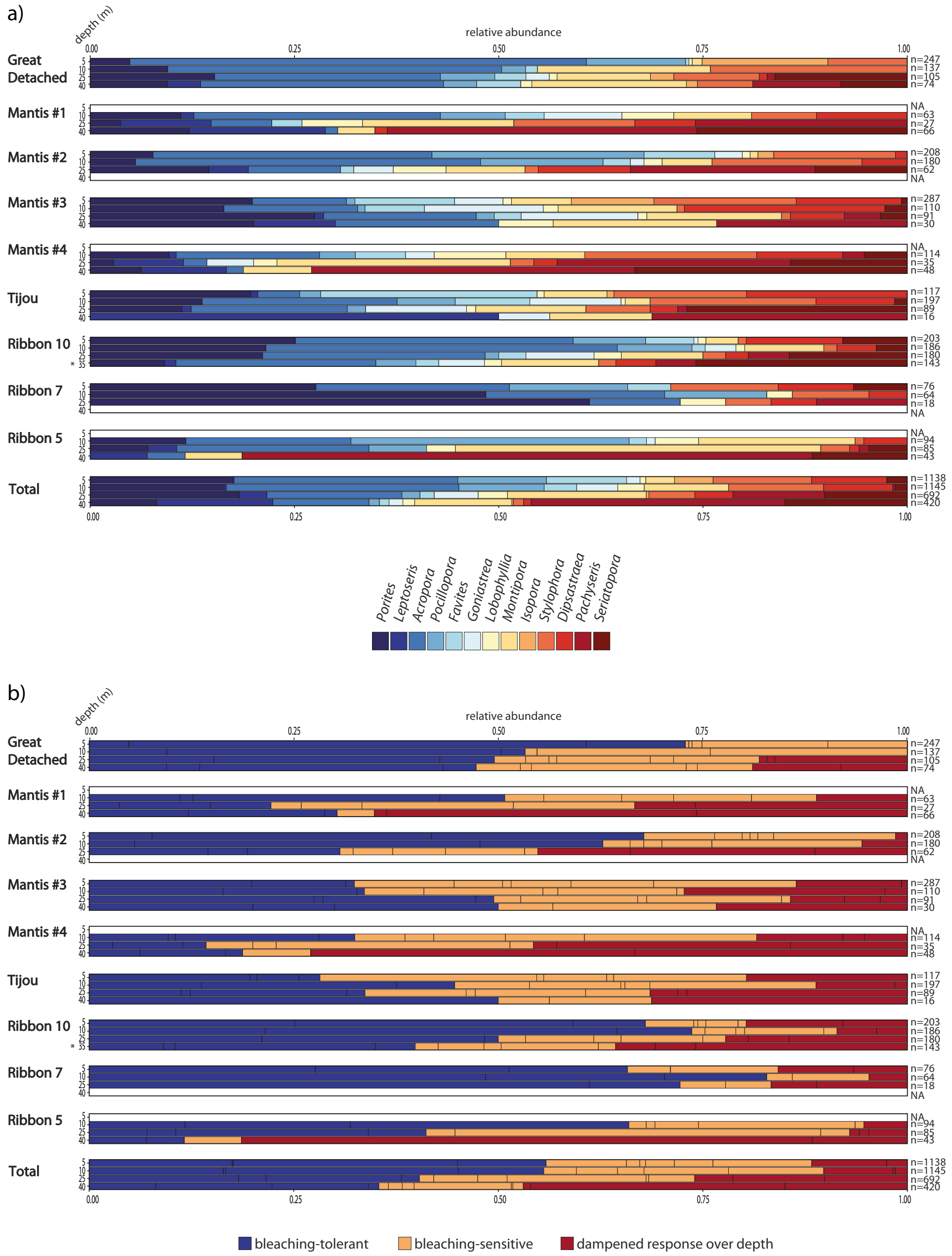

Supplementary Figure 3. Changes in coral community composition across depths for all locations surveyed on the GBR during the mass bleaching event. Stacked bar graphs give, for each depth and location, the relative abundance of **a)** individual coral taxa surveyed at sufficient coverage to be included in the OLR model, and **b)** coral taxa grouped by their relative bleaching susceptibility

as determined by the OLR model. Note that taxa are ordered identically for all stacked bars in panels a) and b). Number of observations noted next to each stacked bar, with NA denoting depths that lacked a coral reef community. (*) Data collected at 35 m depth due to absence of reef formation at 40 m.

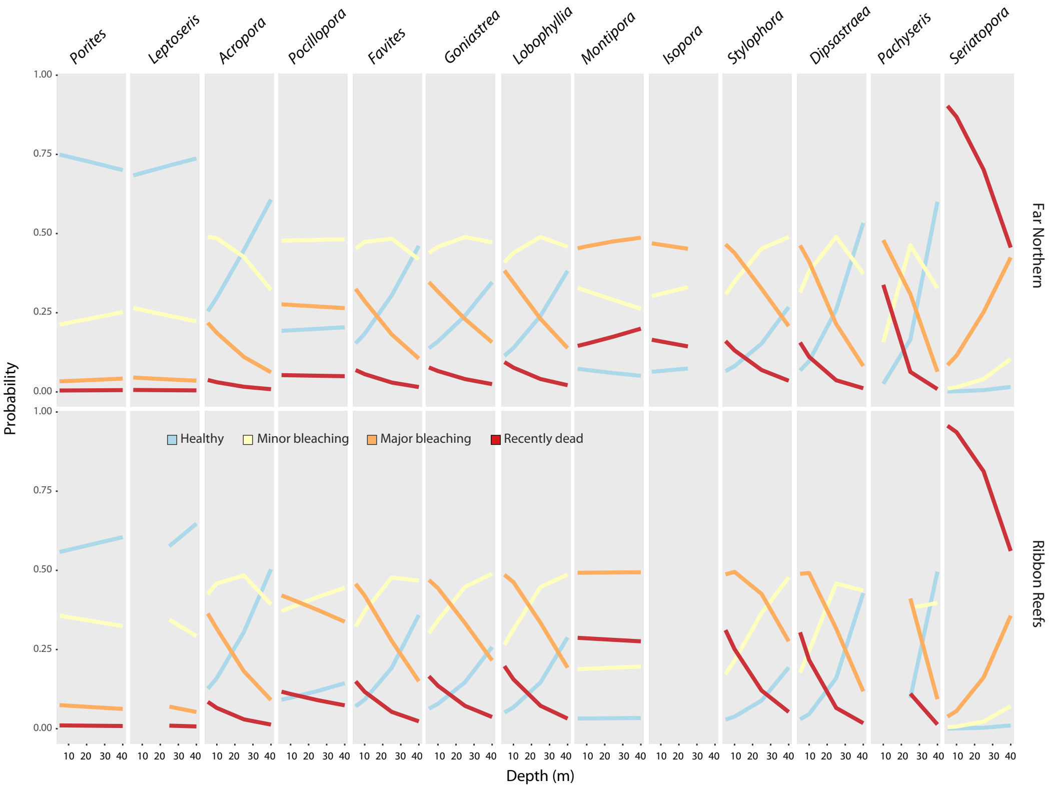

Supplementary Figure 4. Probability values output by the Ordinal Logistic Regression (OLR) model for the different bleaching categories according to the significant explanatory variables (depth, coral taxa and region) and their significant interactions. Inclusion of explanatory variables and their interactions in the OLR model was determined by a forward and backward model selection tool based on the Akaike Information Criteria. The OLR model was then validated by a nested model approach using Chi-square statistics for the main variables depth ($X^2 = 106.75$, d.f. = 1, $p < 2.2x10^{-16}$), region ($X^2 = 83.34$, d.f. = 1, $p < 2.2x10^{-16}$), and coral taxa ($X^2 = 1543.78$, d.f. = 12, $p < 2.2x10^{-16}$), and the interaction effects of depth:taxa ($X^2 = 93.414$, d.f. = 12, $p = 1.074x10^{-14}$), and depth:region ($X^2 = 4.265$, d.f. = 1, $p = 0.03891$). Respective odds ratios given in Figure 5. OLR fitted values show clear differences between bleaching tolerant (e.g., *Porites* and *Leptoseris*) and bleaching susceptible taxa (e.g., *Seriatopora*, *Pachyseris*, *Dipsastraea*, *Stylophora*, *Isopora*, *Montipora*, *Lobophyllia*, *Goniastrea* and *Favites*),

but also clear differences between taxa experiencing a strong effect of depth on their bleaching response (e.g., *Pachyseris* and *Dipastraea*) and those with no evident depth effect (e.g., *Porites*, *Pocillopora*, *Montipora* and *Leptoseris*).

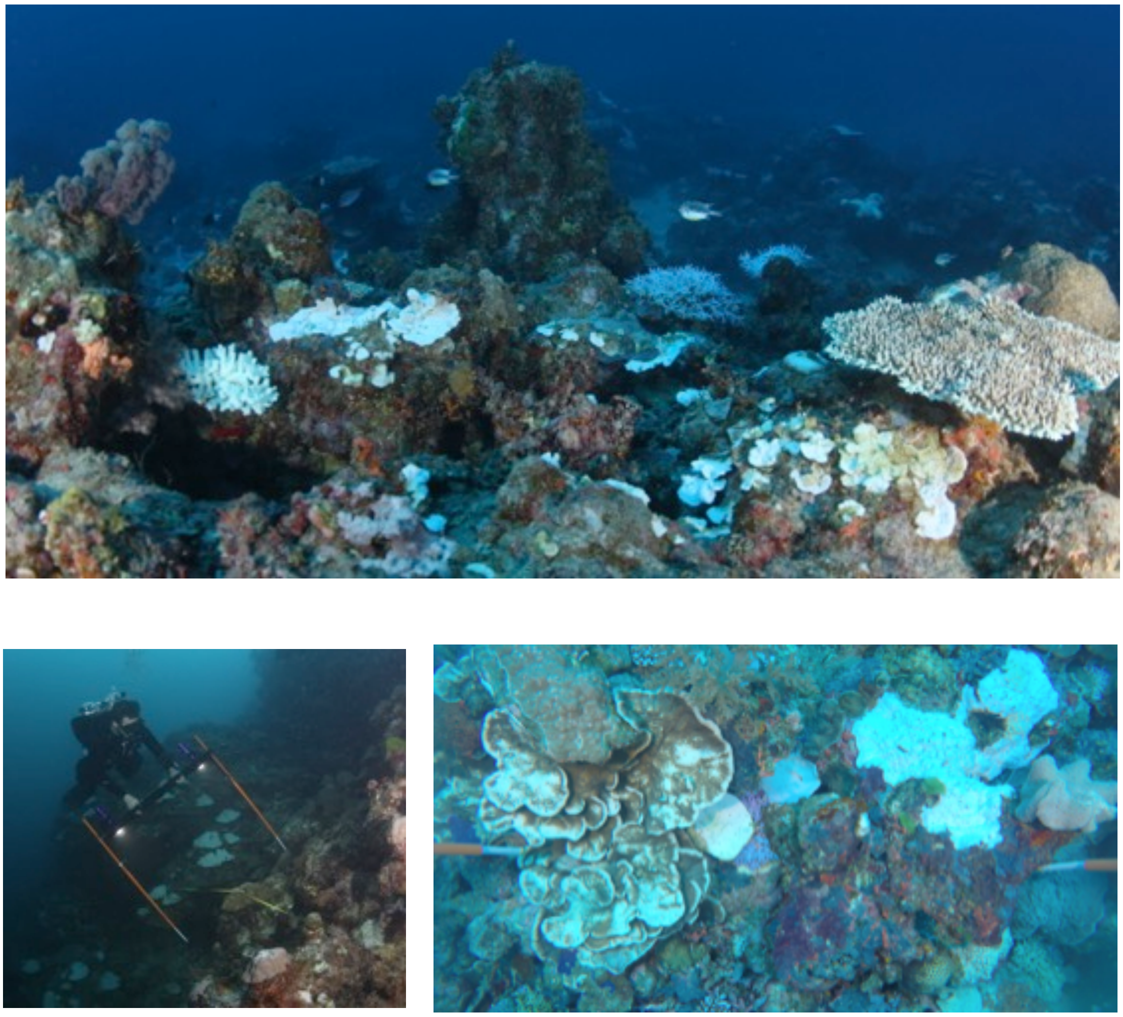

Supplementary Figure 5. Photo examples. a) Ribbon Reef #10 at 35 m depth with many severely bleached coral colonies including *Stylophora* sp., *Montipora* sp. and *Seriatopora* sp., **b)** transect method used to score bleaching impact, note a distance of 1m between the two poles protruding out of the video frame held by the diver (photo taken on Ribbon Reef #10 at 25 m depth), and **c)** video still of shallow bleaching (10 m depth) within transect (with the poles marking the 1m-wide swath). All photographs by Pim Bongaerts and licensed under CC BY-SA 4.0. All rights reserved.

Supplementary References

1 Pickard, G. L., Donguy, J. R., Hennin, C. & Rougerie, F. *A review of the physical oceanography of the Great Barrier Reef and Western Coral Sea.* Vol. 2 (Australian Institute of Marine Science, 1977).

2 Wolanski, E. *Physical oceanographic processes of the Great Barrier Reef.* (CRC Press, 1994).

3 Hughes, T. P. *et al.* Global warming and recurrent mass bleaching of corals. *Nature* **543**, 373-377, doi:10.1038/nature21707 (2017).

4 Frade, P. R., Bongaerts, P., Winkelhagen, A. J. S., Tonk, L. & Bak, R. P. M. In situ photobiology of corals over large depth ranges: A multivariate analysis on the roles of environment, host, and algal symbiont. *Limnol Oceanogr* **53**, 2711-2723 (2008).

5 Glynn, P. W. Coral reef bleaching: Facts, hypotheses and implications. *Global Change Biology* **2**, 495-509, doi:DOI 10.1111/j.1365-2486.1996.tb00063.x (1996).
